# The prognostic value of external vs internal pancreatic duct stents after pancreaticoduodenectomy in patients with FRS ≥ 4: a retrospective cohort study

**DOI:** 10.1186/s12893-021-01074-w

**Published:** 2021-02-12

**Authors:** Yuancong Jiang, Qin Chen, Yi Shao, Zhenzhen Gao, Ming Jin, Bingqiang Gao, Bo Zhou, Sheng Yan

**Affiliations:** 1 Division of Hepatobiliary and Pancreatic Surgery, Department of Surgery, The Second Affiliated Hospital, School of Medicine, Zhejiang University, 88 Jiefang Road, Hangzhou, 310009 China; 2 Cancer Institute (Key Laboratory of Cancer Prevention and Intervention, China National Ministry of Education, Key Laboratory of Molecular Biology in Medical Sciences, Zhejiang Province, China), The Second Affiliated Hospital, School of Medicine, Zhejiang University, Hangzhou, China; 3 Division of Hepatobiliary and Pancreatic Surgery, Department of Surgery, The First Affiliated Hospital, College of Medicine, Zhejiang University, Hangzhou, China

**Keywords:** Clinically relevant postoperative pancreatic fistula (CR-POPF), Pancreaticoduodenectomy, External pancreatic duct stent, Postoperative complications, Prognosis

## Abstract

**Background:**

The prognostic value of external vs internal pancreatic duct stents after pancreaticoduodenectomy remains controversial. This study aimed to evaluate the benefits of external and internal stents using the Fistula Risk Score system with regard to the incidence of clinically relevant postoperative pancreatic fistula.

**Methods:**

A total of 382 patients who underwent pancreaticoduodenectomy with duct to mucosa pancreaticojejunostomy were retrospectively enrolled from January 2015 to October 2019. The receiver operating characteristic curve was performed for subgroup analysis of the patients at different levels of risk for pancreatic fistula.

**Results:**

There were no significant differences in terms of pancreatic fistula or other postoperative complications. According to the receiver operating characteristic curve threshold of 3.5, 172 patients with a Fistula Risk Score ≥ 4 and 210 patients with a Fistula Risk Score < 4 were divided into separate groups. The number of valid cases was insufficient to support the subsequent research in patients with a Fistula Risk Score < 4. In patients with a Fistula Risk Score ≥ 4, the use of an external pancreatic duct stent was significantly more effective than the use of an internal stent, especially with regard to the risk for pancreatic fistula (Grade C) (*P* = 0.039), at ameliorating the incidence of clinically relevant postoperative pancreatic fistula (*P* = 0.019). Additionally, the incidence of lymphatic leakage was significantly higher in the external stent group compared with the internal stent group (*P* = 0.040).

**Conclusions:**

Compared with internal stents, the use of an external stent could reduce the incidence of clinically relevant postoperative pancreatic fistula in patients with a Fistula Risk Score ≥ 4. More large-scale prospective clinical trials are warranted to further clarify our results.

## Background

Pancreatic cancer, with its associated poor prognosis, is one of the most insidious and lethal cancers globally[1]. Indeed, pancreatic cancer has been listed as the fourth leading cause of cancer-related deaths in developed countries, and it may replace colorectal cancer as the second-leading cause of cancer-related deaths by 2030[2, 3]. Pancreaticoduodenectomy is the standard treatment for periampullary carcinoma, especially pancreatic head tumors[4]. However, the incidence of postoperative complications, especially postoperative pancreatic fistula, remains as high as 25%-50%, which limits the dissemination of pancreaticoduodenectomy[5].

An external pancreatic duct stent is one of the methods used to prevent pancreatic fistula. A large number of studies, including prospective randomized trials as well as meta-analyses, have shown that external pancreatic duct stents significantly decrease the rate of pancreatic fistula and shorten the length of hospital stay[6–8]. Paradoxically, several studies have shown that external pancreatic duct stents have no effect and may even increase the incidence of postoperative pancreatic fistula[9–11]. In a meta-analysis, Dong et al. [12] observed that the use of an external pancreatic duct stent was associated with a significantly lower incidence of pancreatic fistula in patients at high risk for pancreatic fistula compared with an internal stent, but there was no definitive conclusion because of the low quality of the evidence[8].

In 2013, Callery et al. [13] proposed the Fistula Risk Score (FRS) according to the International Study Group of Pancreatic Surgery (ISGPS), which quantitatively validated the risk of pancreatic fistula and assessed the benefits of pancreatic duct stents in patients at different levels of risk. Moreover, ISGPS redefined the classification criteria for pancreatic fistula (Grade A) as a biochemical leak, which had no significant clinical impact on the clinical prognosis[14]. In addition, the position statement by ISGPS indicated that, due to the lack of high-quality evidence, the pancreatic duct stent was not routinely recommended during pancreaticoenteric anastomosis, but external stenting can be considered in high-risk glands[15]. Therefore, it is necessary to systematically re-investigate the safety and effectiveness of external pancreatic duct stents for the prevention and treatment of clinically relevant postoperative pancreatic fistula (CR-POPF) in both high-risk and low-risk patients. This retrospective study was conducted to evaluate the differences between external and internal pancreatic stents using the FRS scoring system, based on the hypothesis that the use of an external stent in high-risk patients could decrease the rates of CR-POPF compared with an internal stent after pancreaticoduodenectomy.

## Methods

### Patients

Clinical data of 382 patients who underwent pancreaticoduodenectomy in our pancreatic center from January 2015 to October 2019 were retrospectively collected. None of the patients had a past history of abdominal surgery or other cancers which might cause intra-abdominal adhesion. Patients who cannot perform pancreaticoduodenectomy due to severe comorbidities associated with the heart, lungs, liver, kidneys or organ metastases were excluded. Next, using the optimum cutoff point of the receiver operating characteristic (ROC) curve for subgroup analysis, these patients were separated into two groups: those at relatively high-risk for pancreatic fistula or those at relatively low-risk for pancreatic fistula.

### Surgical technique

All pancreaticoduodenectomies were performed by the same surgical team at our pancreatic center throughout the study period. According to the preoperative laboratory results, medical imaging data and intraoperative conditions, the laparoscopic or open pancreaticoduodenectomy, or pylorus-preserving pancreaticoduodenectomy was performed at the discretion of the individual surgeon. Child’s technique was implemented to achieve digestive tract reconstruction, and all patients underwent a duct-to-mucosa pancreaticojejunostomy. Furthermore, the internal pancreatic duct stent, a silicone catheter with multiple side pores, was inserted into the main pancreatic duct and the other end was placed in the jejunum cavity. Alternately, the external pancreatic duct stent left the other end exteriorized through the proximal jejunum via a small enterotomy that was fixed in the abdominal wall. All pancreaticoduodenectomies at our pancreatic center were implemented with external pancreatic duct stent or internal stent based on the experiences of the surgeons. Fibrin glue sealant was not applied during pancreaticojejunostomy anastomosis, and two abdominal drainage tubes were placed, one behind the biliary-enteric anastomosis and the other around the pancreatic-enteric anastomosis.

### Perioperative management

Each patient underwent standardized perioperative management at the Second Affiliated Hospital, College of Medicine, Zhejiang University. Broad-spectrum antibiotics and anti-anaerobic drugs were used 72 h postoperatively to prevent infection after surgery. Jejunal-placed feeding tubes, nasogastric tubes and octreotide were routinely used. After bowel sounds were restored, the nasogastric tube was removed, and the normal diet was gradually resumed after 3–5 days.

Amylase levels in the serum and drainage fluid were routinely measured on postoperative days 3, 5 and 7. Then, a contrast-enhanced computed tomography was performed on postoperative days 5–7 to screen for any possible complications. If there was no evidence of complications such as pancreatic fistula, bile leakage or infection, the abdominal drainage tube and external pancreatic duct stent were gradually removed during hospitalization. If a pancreatic fistula, a severe abdominal infection, or other severe complication was found, antibiotics were used based on blood cultures and drug sensitivity tests until the serum and drainage fluid amylase returned to normal levels and the external pancreatic duct stent was left in situ when the patient was discharged. One month after surgery, the external pancreatic duct stent was removed in the outpatient clinic.

### Study endpoints

The primary endpoint of this retrospective study was the incidence of CR-POPF, which was graded according to the definition set forth by ISGPS in 2016[14]. The risk of CR-POPF was evaluated by FRS[13]. The secondary endpoints of this retrospective study included the incidence of delayed gastric emptying (DGE), postoperative pancreatic hemorrhage (PPH), bile leakage, infection and mortality, which were graded according to the Clavien-Dindo Classification[16]. The incidence of complications was collected from medical records and a one-month outpatient follow-up period was implemented for patients with complications at the time of discharge.

### Statistical analysis

SPSS 25.0 (International Business Machines Corporation, Armonk, New York) was used for all statistical analyses. The sample size was calculated using the Epi-Info companion. All continuous data were tested for normality using the Kolmogorov–Smirnov test. Normally distributed data were expressed as the mean ± standard deviation and were analyzed using the unpaired, independent, two-tailed t-test. The other continuous data were expressed in interquartile ranges, and two samples rank sum tests were used. All categorical variables were tested using the chi-square test. Variables with *P* values less than 0.05 in the univariate analysis were included in the multivariate analysis as independent risk factors. *P* < 0.05 was considered statistically significant.

## Results

Between January 2015 and October 2019, a total of 382 patients underwent pancreaticoduodenectomy and were enrolled in this study. Two hundred and ten patients had an FRS score of less than 4, 163 patients had an FRS score of 4–6, and 9 patients had an FRS score of greater than 7. Forty-five patients received an external pancreatic duct stent, and 337 patients received an internal pancreatic duct stent. There were no significant differences with regard to age (*P* = 0.202), gender (*P* = 0.734), body mass index (BMI) (*P* = 0.072), hypertension (*P* = 0.592), smoking status (*P* = 0.948), drinking (*P* = 0.057), weight loss (*P* = 0.292), intraoperative bleeding (*P* = 0.214) or pathological diagnosis (*P* = 0.428) between the two groups. Compared to the internal stent group, the external stent group had a higher proportion of patients with a soft pancreas parenchyma (*P* < 0.001) and a small pancreatic duct diameter (*P* < 0.001), although there was a smaller proportion of patients with diabetes (*P* = 0.015). However, the incidence of pancreatic fistula or CR-POPF did not differ between the external and internal stent groups. The patient characteristics and postoperative complications are listed in Table 1 and the flow diagram for the study is shown in Additional file 1: Figure S1.

**Table 1:** Baseline characteristics of external stent and internal stent group in all patients

Characteristics	External stent group N = 45	Internal stent group N = 337	*P*-value
Age (years)	60 (54–67)	64 (56–70)	*P* = 0.202
Gender (M/F)	27/18	211/126	*P* = 0.734
BMI (kg/m^2^)	23.01 ± 3.14	22.17 ± 2.93	*P* = 0.072
Hypertension	12 (26.7%)	103 (30.6%)	*P* = 0.592
Diabetes	1 (2.2%)	53(15.7%)	*P = 0.015*
Smoke	17 (37.8%)	129 (38.3%)	*P* = 0.948
Drink	9 (20.0%)	115 (34.1%)	*P* = 0.057
Weight loss	8 (17.8%)	84 (24.9%)	*P* = 0.292
Operative time (min)	496.93 ± 109.06	425.39 ± 142.77	*P = 0.001*
Intraoperative bleeding (mL)	275 (200–400)	200 (200–300)	*P* = 0.214
Texture soft/Firm	43/2	179/158	*P < 0.001*
Pancreatic duct diameter (cm)	0.3 (0.3–0.4)	0.4 (0.3–0.5)	*P < 0.001*
Pathology			*P* = 0.428
Pancreatic adenocarcinoma or pancreatitis	15 (33.3%)	133 (39.5%)	
Ampullary, duodenal, cystic, islet cell, etc	30 (66.7%)	204 (60.5%)	
Pancreatic fistula	22 (48.9%)	128 (38.0%)	*P* = 0.159
CR-POPF	8 (17.8%)	68 (20.2%)	*P* = 0.705
Grade A	14 (31.1%)	60 (17.8%)	
Grade B	8 (17.8%)	55 (16.3%)	
Grade C	0 (0%)	13 (3.9%)	*P* = 0.379

The italic values reflected that there was a significant difference between the two groups

Considering that an external pancreatic duct stent is always used in patients at higher risk for pancreatic fistula, the ROC curve was used to find the threshold of the FRS. When FRS = 3.5, the Youden’s index is the highest, and the area under the curve (AUC) is 0.721. The ROC curve is shown in Additional file 2: Figure S2. Therefore, a total of 172 patients with FRS ≥ 4 were included in the subsequent research regarding the effect of an external stent in patients at relatively high-risk for pancreatic fistula. For the 210 patients with FRS < 4, there were only 2 patients who received an external pancreatic duct stent. Concretely, a total of 19 patients with FRS < 4 developed CR-POPF (8.6%), with 0 patients in the external stent group (0.0%) and 19 patients in the internal stent group (9.1%). The number of valid cases was, therefore, insufficient to support the subsequent comparison of external stents and internal stents in patients at relatively low-risk for pancreatic fistula.

In the subgroup analysis of patients with FRS ≥ 4, 43 patients received an external pancreatic duct stent and 129 patients received an internal pancreatic duct stent. There were no significant differences with regard to the baseline characteristics between the two groups except for diabetes. Compared with patients in the internal stent group, the proportion of patients with diabetes in the external stent group was lower (*P* = 0.028). The characteristics of the patients at high-risk for a pancreatic fistula are listed in Table 2. The pancreaticoduodenectomy is confounded by many factors that affect the risk of pancreatic fistula, so we controlled for the anastomosis technique and the treatments that may be used to prevent pancreatic fistula, such as octreotide and fibrin glue sealant, to reduce subjective bias. As shown in Table 3, in contrast to the internal stent group, the external stent group had longer operation duration (492.16 ± 103.63 vs 432.22 ± 136.51, *P* = 0.003), more combined vascular resections (18.6% vs 3.9%, *P* = 0.004), and a higher proportion of patients with pancreatic cancer or chronic pancreatitis (*P* = 0.002).

**Table 2:** Baseline characteristics of external stent and internal stent group in high-risk of pancreatic fistula patients

Characteristics	External stent group N = 43	Internal stent group N = 129	*P*-value
Age (years)	60 (56–67)	62 (56–70)	*P* = 0.406
Gender (M/F)	27/16	84/45	*P* = 0.783
BMI (kg/m^2^)	23.03 ± 3.18	22.53 ± 2.79	*P* = 0.334
Hypertension	11 (25.6%)	39 (30.2%)	*P* = 0.561
Diabetes	1 (2.3%)	19 (14.7%)	*P = 0.028*
Smoke	16 (37.2%)	47 (36.4%)	*P* = 0.927
Drink	8 (18.6%)	37(28.7%)	*P* = 0.193
Weight Loss	8 (18.6%)	23 (17.8%)	*P* = 0.909

The italic values reflected that there was a significant difference between the two groups

**Table 3:** Comparison of intraoperative variables between external stent and internal stent group in high-risk of pancreatic fistula patients

Variables	External stent group N = 43	Internal stent group N = 129	*P*-value
Preoperative biliary drainage	12 (27.9%)	27 (20.9%)	*P* = 0.344
Percutaneous/endoscopic	7/5	7/20	
Preoperative total bilirubin (μmol/L)	17.5 (11–135)	28 (13.5–121.5)	*P* = 0.581
Preoperative direct bilirubin (μmol/L)	9.9 (4–107.3)	9.7 (3–79.7)	*P* = 0.535
Preoperative CA 199 (U/mL)	20.7 (5.8–191)	27 (7.9–156.2)	*P* = 0.965
Operative time (min)	492.16 ± 103.63	432.22 ± 136.51	*P = 0.003*
Intraoperative bleeding (mL)	225 (200–400)	200 (200–400)	*P* = 0.712
≤ 400	33	101	
> 400	10	28	
Vascular resection	8 (18.6%)	5 (3.9%)	*P = 0.004*
Blood transfusion	17 (39.5%)	38 (29.5%)	*P* = 0.220
Texture soft/Firm	43/0	121/8	*P* = 0.203
Pancreatic duct diameter (cm)	0.3(0.3–0.4)	0.3 (0.3–0.3)	*P* = 0.205
0.1	1	0	
0.2	6	26	
0.3	22	76	
0.4	12	22	
0.5	2	4	
> 0.5	0	1	
Pathology			*P = 0.002*
Pancreatic adenocarcinoma or pancreatitis	13	14	
Ampullary, duodenal, cystic, islet cell, etc	30	115	
Pancreatic adenocarcinoma	12	12	
Cholangiocarcinoma	2	16	
Duodenal carcinoma	16	39	
Chronic pancreatitis	1	2	
Pancreatic cystic tumor	1	19	
Pancreatic neuroendocrine tumor	1	3	
Ampullary carcinoma	5	26	
Others	5	12	

The italic values reflected that there was a significant difference between the two groups

The overall complication rate was 27/43 (62.7%) in the external stent group and 79/129 (61.2%) in the internal stent group. Pancreatic fistula was the most common complication in our retrospective study. A total of 57 patients developed CR-POPF (33.1%), with 8 patients in the external stent group and 49 patients in the internal stent group. External pancreatic duct stents were significantly more effective than internal stents at ameliorating CR-POPF (38.0% vs 18.6%, *P* = 0.019). Notably, the incidence of pancreatic fistula (Grade C) in the external stent group was significantly lower than in the internal stent group (0.0% vs 9.3%, *P* = 0.039). Additionally, 46 patients experienced an abdominal infection (26.7%), and while the incidence of abdominal infection in the external stent group was lower than in the internal stent group (20.9% vs 28.7%, *P* = 0.320), the difference was not statistically significant. Twenty-eight patients experienced lymphatic leakage (16.3%), including 11 patients in the external stent group and 16 patients in the internal stent group. Lymphatic leakage in the external stent group tended to be significantly greater than in the internal stent group (25.6% vs 12.4%, *P* = 0.040).

The incidence of other complications was low. A total of 27 patients experienced PPH (15.7%), 21 patients required additional surgery (12.2%), 16 patients contracted pulmonary infections (9.3%), 14 patients were found to have DGE (8.1%), 11 patients developed pancreatic pseudocysts (6.4%), 9 patients experienced bile leakage (5.2%), 9 patients died during hospitalization (5.2%), 7 patients developed intestinal obstruction (4.1%) and stent-related complications occurred in 3 patients (1.7%). No statistically significant difference in complications was found between the two groups. The postoperative complications between the external and internal stent groups are listed in Table 4.

**Table 4:** Comparison of postoperative complications between external stent and internal stent group in high-risk of pancreatic fistula patients

Postoperative complications	External stent group N = 43	Internal stent group N = 129	*P*-value
Pancreatic fistula	22 (51.2%)	78 (60.5%)	*P* = 0.284
CR-POPF	8 (18.6%)	49 (38.0%)	*P = 0.019*
Grade A	14 (32.6%)	29 (22.5%)	*P* = 0.186
Grade B	8 (18.6%)	37 (28.7%)	*P* = 0.193
Grade C	0 (0.0%)	12 (9.3%)	*P = 0.039*
Delayed gastric emptying	3 (7.0%)	11 (8.5%)	*P* = 1.000
Intra-abdominal bleeding	6 (14.0%)	21 (16.3%)	*P* = 0.717
Lymphatic leakage	11 (25.6%)	16 (12.4%)	*P = 0.040*
Bile leakage	4 (9.3%)	5 (3.9%)	*P* = 0.230
Intestinal obstruction	2 (4.7%)	5 (3.9%)	*P* = 1.000
Pancreatic pseudocyst	3 (7.0%)	8 (6.2%)	*P* = 1.000
Pulmonary infection	3 (7.0%)	13 (10.1%)	*P* = 0.764
Abdominal infection	9 (20.9%)	37 (28.7%)	*P* = 0.320
Re-operation	5 (11.6%)	16 (12.4%)	*P* = 0.893
In-hospital mortality	1 (2.3%)	8 (6.2%)	*P* = 0.453
Stent-related complications	2 (4.7%)	1 (0.8%)	*P* = 0.155
Postoperative hospital stay (day)	18 (14–25)	17 (13–26)	*P* = 0.715

The italic values reflected that there was a significant difference between the two groups

Univariate analysis revealed that BMI (*P* = 0.009), hypertension (*P* = 0.009), pancreatic duct stent (*P* = 0.022), blood transfusion (*P* = 0.020) and pancreatic duct diameter (*P* = 0.014) were identified as significant risk factors for CR-POPF. Next, multivariate analysis was used to explore the independent risk factors for CR-POPF after pancreaticoduodenectomy. As shown in Table 5, high BMI (OR 1.175, *P* = 0.013), the use of an internal stent (OR 3.376, *P* = 0.011), receipt of a blood transfusion (OR 2.777, *P* = 0.008) and a narrow pancreatic duct (OR 0.003, *P* = 0.024) were independent risk factors predictive of CR-POPF.

**Table 5:** Univariable and multivariable analysis of risk factors for clinically relevant postoperative pancreatic fistula in high-risk of pancreatic fistula patients

	Univariable analysis			Multivariable analysis		
	OR	95%CI	*P*-value	OR	95%CI	*P*-value
Age (years)	1.000	0.969–1.032	0.999			
Female (ref: male)	0.976	0.502–1.896	0.942			
BMI (kg/m^2^)	1.167	1.040–1.311	*0.009*	1.175	1.034–1.335	*0.013*
Hypertension	2.490	1.257–4.932	*0.009*	1.791	0.838–3.829	0.133
Diabetes	2.234	0.871–5.728	0.094			
Smoke	0.718	0.366–1.406	0.334			
Drink	0.663	0.312–1.409	0.285			
Weight Loss	1.262	0.847–1.880	0.253			
Preoperative biliary drainage	1.354	0.645–2.843	0.423			
Preoperative total bilirubin (μmol/L) (ref: ≤ 21)	1.415	0.738–2.715	0.296			
Preoperative direct bilirubin (μmol/L) (ref: ≤ 8)	1.613	0.848–3.069	0.145			
Preoperative CA 199 (U/mL) (ref: ≤ 37)	0.791	0.416–1.506	0.476			
Operative time (min)	0.999	0.997–1.002	0.463			
Intraoperative bleeding (mL)	1.000	0.999–1.001	0.815			
Vascular resection	0.583	0.154–2.208	0.427			
Internal stent (ref: external stent)	2.680	1.149–6.247	*0.022*	3.376	1.318–8.646	*0.011*
Blood transfusion	2.214	1.134–4.319	*0.020*	2.777	1.310–5.887	*0.008*
Texture Firm (ref: soft)	0.276	0.033–2.295	0.233			
Pancreatic duct diameter (cm)	0.003	0.000–0.319	*0.014*	0.003	0.000–0.456	*0.024*
Pancreatic adenocarcinoma or pancreatitis (ref: ampullary, duodenal, cystic, islet cell, etc.)	1.480	0.636–3.440	0.363			

The italic values reflected that there was a significant difference between the two groups

## Discussion

Pancreatic fistula is one of the most common and serious complications after pancreaticoduodenectomy and may lead to potentially fatal complications. In 1999, Roder et al. [7] reported that the use of external pancreatic duct stents could reduce the incidence of pancreatic fistula from 29.3% to 6.8% and shorten the length of hospital stay, which demonstrated the capacity of external pancreatic duct stents to be an effective method for pancreatic fistula prevention. The reasons for the current use of external stents for the treatment of pancreatic fistula are as follows: First, the patency of the pancreatic duct is supported by external pancreatic stents so that the pancreatic enzymes that accumulate in the jejunum after surgery can be expelled expediently, avoiding activation by enterokinase, and therefore preventing further digestion of the residual pancreas. Second, the successful discharge of pancreatic juices relieves the tension on the pancreatic-enteric anastomosis and promotes postoperative anastomotic healing. Moreover, the use of external pancreatic duct stents allows for a more precise placement of the sutures during pancreaticoduodenectomy to avoid suture injury and further iatrogenic damage[6, 7, 17]. Compared with simply stenting the pancreatic duct into the jejunum cavity, the external stent minimizes the digestive erosion of the residual pancreas and fully eliminates pancreatic juices from the anastomosis. In addition, the volume of pancreatic juice drainage can be routinely measured as a precaution against premature detachment of the pancreatic duct stent following pancreaticojejunostomy. Whereas, DGE may result from the loss of a large amount of fluid and digestive enzymes, and better nutritional support and volume maintenance is therefore required. Additionally, compared with internal stents, external stents are more likely to cause stent-related complications, such as bending, displacement, shedding, or blockage of the drainage tube, as well as peritonitis, chronic pancreatitis or stenosis after removal of the stent[18, 19]. Thus, the benefit of an external over an internal stent for the prevention of CR-POPF remains controversial[10, 20, 21].

Based on the new definition of pancreatic fistula and the lack of explicit selectivity with validated FRS for high-risk patients receiving an external pancreatic duct stent in the current literature, some patients at low risk of pancreatic fistula may not benefit from an external stent, resulting in insufficient results. According to the ROC curve, the FRS threshold was found to be 3.5. Thus, in order to compare the efficacy of external pancreatic duct stents with internal stents in the prevention and treatment of CR-POPF, we combined the FRS system to quantitatively evaluate the effect of the two types of pancreatic stents on patients at different levels of risk for pancreatic fistula. Eventually, 172 patients with FRS ≥ 4 were enrolled to evaluate the differences between external and internal stents.

Our retrospective study provided two important findings. First, for patients at high-risk of pancreatic fistula with FRS ≥ 4, the use of an external stent was associated with a lower rate of CR-POPF, which is consistent with the statement by the ISGPS. Moreover, external stents exerted a more pronounced effect than the internal stents on the prevention of pancreatic fistula (Grade C). No pancreatic fistula (Grade C) and only 8 pancreatic fistulas (Grade B) occurred in the 43 patients who received an external stent. Interestingly, there was no significant difference in the overall incidence of pancreatic fistula between the two groups, and the proportion of patients with pancreatic fistula (Grade A) was even higher in the external stent group than in the internal stent group, which may explain the uncertain results in the previous studies. Second, compared with the patients who received an internal stent, the incidence of lymphatic leakage in patients with external pancreatic duct stents was significantly increased. Due to the higher proportion of patients with pancreatic cancer in the external stent group, more vascular resections and more extensive lymph node dissections were required to achieve curative (R0) resection, which meant that patients who received external stenting required longer hospital stays for the conservative treatment of lymphatic leakage and subsequent complications, such as electrolyte disturbances or abdominal infections. Collectively, the association between pancreatic stent and lymphatic leakage was not clear, but it appeared related to the pathology type and extension of the operation rather than the presence of an external stent.

Although our retrospective study indicated that the use of an external pancreatic duct stent has an excellent preventive effect against CR-POPF, especially pancreatic fistula (Grade C) in patients with FRS ≥ 4, there remain some limitations in our research. First, due to the retrospective design without randomized allocation, there may have been some bias in the selection of the controls and baseline characteristics between external stent and internal stent group in all patients or in high-risk pancreatic fistula patients. This bias may be caused by the discretion of the individual surgeon which may have an impact on the outcome. In clinical practice, the external stent is more likely to be used in patients with soft pancreatic parenchyma and small pancreatic ducts. As for the bias of diabetes, a subgroup analysis was performed to resolve the selection bias. As shown in the Additional file 3: Table S1 and Additional file 4: Table S2, consistent results were observed, with favorable outcomes of external stents in patients without diabetes. However, the cases were not valid to support the subsequent comparison between external stents vs internal stents in patients with diabetes. Besides, to reduce the selection bias and enhance the accuracy of our research, we conducted a rigorous control of the technique of anastomosis and application of octreotide or other measures, and standardized perioperative management was performed according to our hospital’s policies. However, the outcomes following placement of the two stents were still impacted by the surgeons’ clinical experience and subjective choices. Second, as the external stent was more frequently used in patients with high FRS scores, the sample size was insufficient to detect a statistically significant effect in patients with FRS < 4. Third, although the FRS system is currently a classic and broadly deployed risk prediction tool for CR-POPF worldwide, it still has its shortcomings. There are many up-to-date scoring systems available, such as the alternative Fistula Risk Score or the Updated Alternative Fistula Risk Score, to validate and further expand the FRS’s scope of application[22]. More large multicenter randomized prospective clinical trials are warranted based on the latest pancreatic fistula risk scoring system to verify the effectiveness of the implementation of external pancreatic duct stents in the prevention and treatment of CR-POPF in patients at different levels of risk for pancreatic fistula.

## Conclusions

The use of external pancreatic duct stents could reduce the incidence of CR-POPF in patients with FRS ≥ 4, but the incidence of lymphatic leakage may be increased. The relationship between external stent placement and lymphatic leakage still needed further investigation. However, it is still recommended that patients with FRS ≥ 4 receive an external stent to avoid the possibly life-threatening occurrence of CR-POPF.

## Supplementary Information

**Additional file 1: Figure S1. **Flow diagram for the study. FRS, Fistula Risk Score.

**Additional file 2: Figure S2.** The ROC curve predicts the FRS score threshold. The area under the ROC curve (AUC) is 0.721. When FRS is 3.5, the sensitivity and specificity are 0.75 and 0.376, respectively. ROC, the receiver operating characteristic curve; FRS, Fistula Risk Score.

**Additional file 3: Table S1. **Baseline characteristics of External stent and Internal stent group in all patients without diabetes.

**Additional file 4: Table S2. **Baseline characteristics of External stent and Internal stent group in high-risk of pancreatic fistula patients without diabetes.

## Data Availability

The data used to support the findings of this study are available from the corresponding author upon reasonable request.

## Abbreviations

FRS: Fistula Risk Score
ISGPS: International Study Group of Pancreatic Surgery
CR-POPF: Clinically relevant postoperative pancreatic fistula
ROC: Receiver operating characteristic
DGE: Delayed gastric emptying
PPH: Postoperative pancreatic hemorrhage
BMI: Body mass index
AUC: Area under the curve
