# Supplementary figures and images for: The prognostic value of external vs internal pancreatic duct stents after pancreaticoduodenectomy in patients with FRS ≥ 4: a retrospective cohort study
Source: BMC Surg. 2021 Feb 12;21:81. doi: 10.1186/s12893-021-01074-w (PMC7881586; doi:10.1186/s12893-021-01074-w)

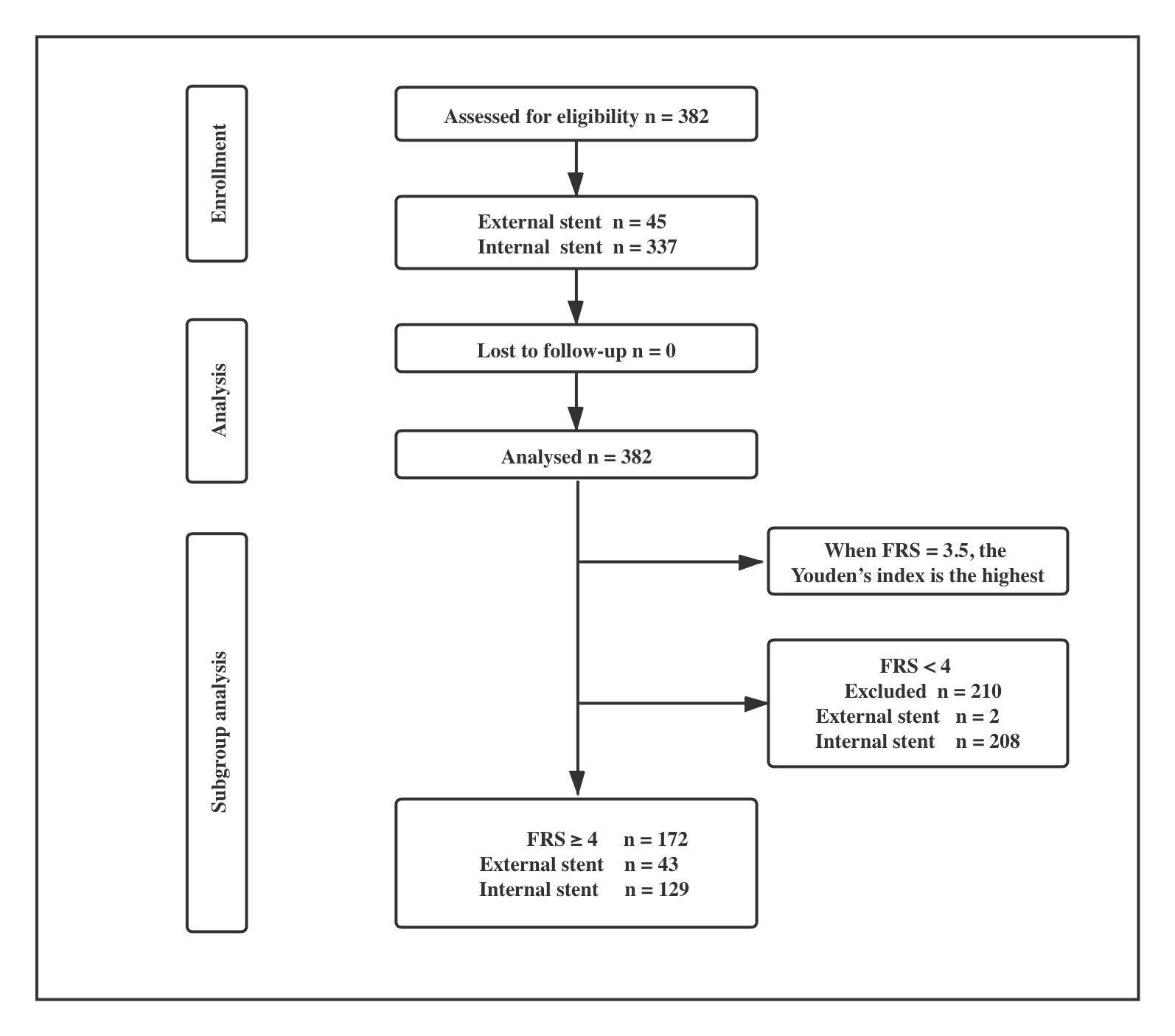

Supplement: Supplementary file 1 — Additional file 1: Figure S1. Flow diagram for the study. FRS, Fistula Risk Score. [file 12893_2021_1074_MOESM1_ESM.tif]

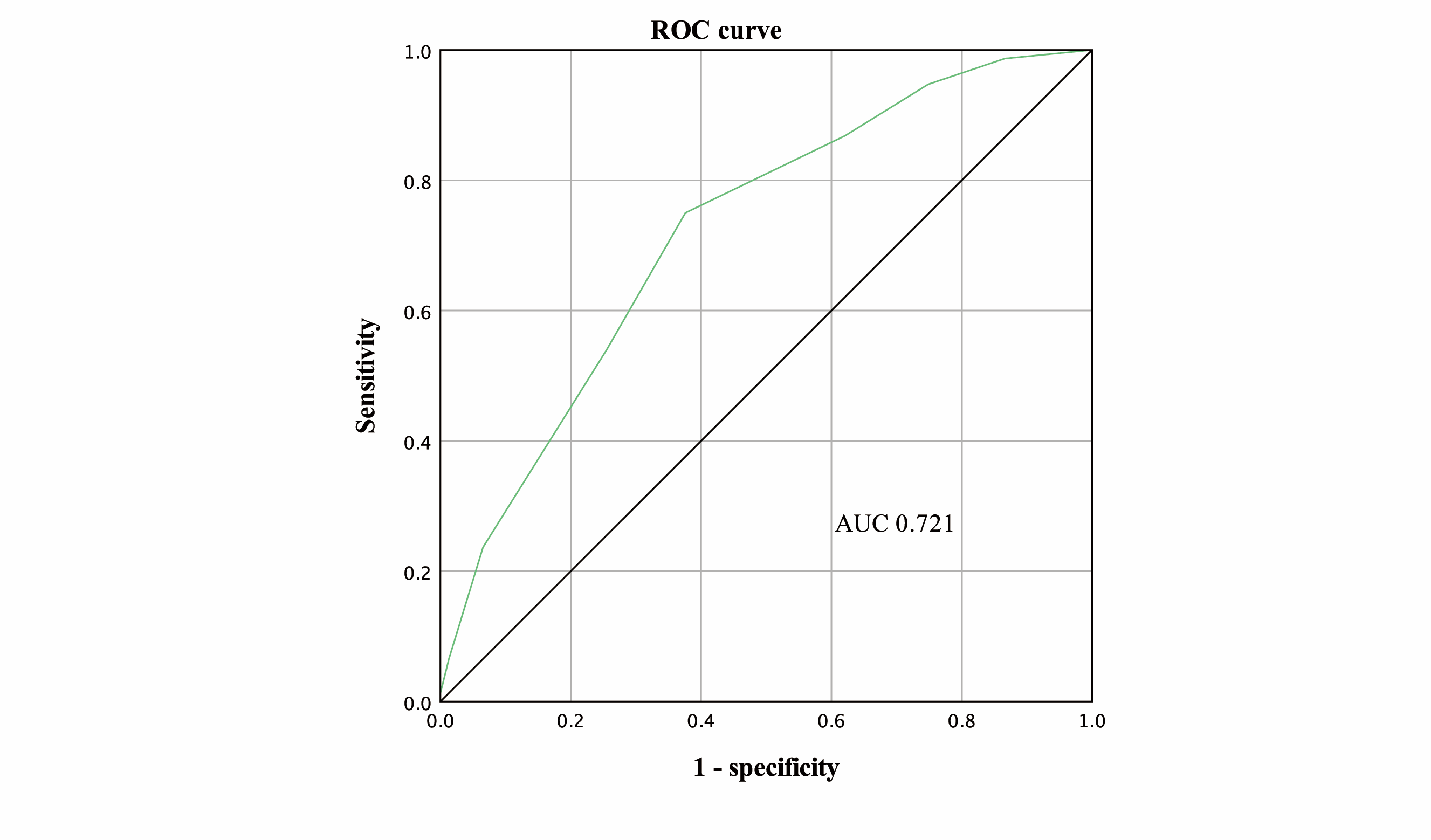

Supplement: Supplementary file 2 — Additional file 2: Figure S2. The ROC curve predicts the FRS score threshold. The area under the ROC curve (AUC) is 0.721. When FRS is 3.5, the sensitivity and specificity are 0.75 and 0.376, respectively. ROC, the receiver operating characteristic curve; FRS, Fistula Risk Score. [file 12893_2021_1074_MOESM2_ESM.tif]
