# Supplementary material for: The prognostic value of external vs internal pancreatic duct stents after pancreaticoduodenectomy in patients with FRS ≥ 4: a retrospective cohort study
Source: BMC Surg. 2021 Feb 12;21:81. doi: 10.1186/s12893-021-01074-w (PMC7881586; doi:10.1186/s12893-021-01074-w)
Supplement: Supplementary file 3 — Additional file 3: Table S1. Baseline characteristics of External stent and Internal stent group in all patients without diabetes. [file 12893_2021_1074_MOESM3_ESM.docx]

TABLE S1. Baseline characteristics of External stent and Internal stent group in all patients without diabetes

| Characteristics | External stent group  N = 44 | Internal stent group  N = 284 | *P* -value |
| --- | --- | --- | --- |
| Pancreatic fistula  CR-POPF  Grade A  Grade B  Grade C | 21(47.7%)  8(18.2%)  13(29.5%)  8(18.2%)  0(0%) | 111(39.1%)  56(19.7%)  55(19.4%)  44(15.5%)  12(4.2%) | *P* = 0.277  *P* = 0.811  *P* = 0.338 |
