# Supplementary material for: The prognostic value of external vs internal pancreatic duct stents after pancreaticoduodenectomy in patients with FRS ≥ 4: a retrospective cohort study
Source: BMC Surg. 2021 Feb 12;21:81. doi: 10.1186/s12893-021-01074-w (PMC7881586; doi:10.1186/s12893-021-01074-w)
Supplement: Supplementary file 4 — Additional file 4: Table S2. Baseline characteristics of External stent and Internal stent group in high-risk of pancreatic fistula patients without diabetes. [file 12893_2021_1074_MOESM4_ESM.docx]

TABLE S2. Baseline characteristics of External stent and Internal stent group in high-risk of pancreatic fistula patients without diabetes

| Characteristics | External stent group  N = 42 | Internal stent group  N = 110 | *P* -value |
| --- | --- | --- | --- |
| Pancreatic fistula  CR-POPF  Grade A  Grade B  Grade C | 21(50.0%)  8(19.0%)  13(31.0%)  8(19.0%)  0(0%) | 66(60.0%)  39(35.5%)  27(24.5%)  28(25.5%)  11(10.0%) | *P* = 0.265  *P =* 0.050  ***P =* 0.033** |
